# A Novel Dynamic Spectrum Access Framework Based on Reinforcement Learning for Cognitive Radio Sensor Networks

**DOI:** 10.3390/s16101675

**Published:** 2016-10-12

**Authors:** Yun Lin, Chao Wang, Jiaxing Wang, Zheng Dou

**Affiliations:** 1 College of Information and Communication Engineering, Harbin Engineering University, Harbin 150001, China; wangchao@hrbeu.edu.cn (C.W.); douzheng@hrbeu.edu.cn (Z.D.); 2 Beijing Huawei Digital Technologies Co., Ltd., Beijing 100032, China; wangjiaxing1@huawei.com

**Keywords:** dynamic spectrum access, control channel, power allocation, reinforcement learning

## Abstract

Cognitive radio sensor networks are one of the kinds of application where cognitive techniques can be adopted and have many potential applications, challenges and future research trends. According to the research surveys, dynamic spectrum access is an important and necessary technology for future cognitive sensor networks. Traditional methods of dynamic spectrum access are based on spectrum holes and they have some drawbacks, such as low accessibility and high interruptibility, which negatively affect the transmission performance of the sensor networks. To address this problem, in this paper a new initialization mechanism is proposed to establish a communication link and set up a sensor network without adopting spectrum holes to convey control information. Specifically, firstly a transmission channel model for analyzing the maximum accessible capacity for three different polices in a fading environment is discussed. Secondly, a hybrid spectrum access algorithm based on a reinforcement learning model is proposed for the power allocation problem of both the transmission channel and the control channel. Finally, extensive simulations have been conducted and simulation results show that this new algorithm provides a significant improvement in terms of the tradeoff between the control channel reliability and the efficiency of the transmission channel.

## 1. Introduction

**Figure 1:**
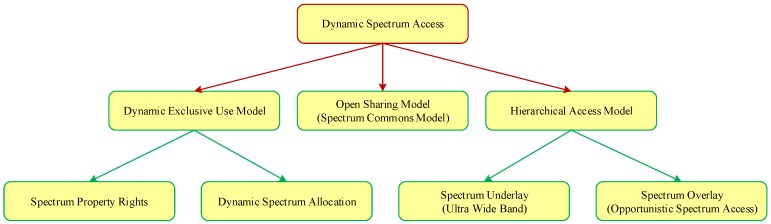
The dynamic spectrum access models.

Cognitive radio (CR) is a promising technology which can fully use the spectrum by dynamically accessing the primary network. Consequently, dynamic spectrum access technology plays a very significant role and has become a hot research topic. As illustrated in Figure 1, dynamic spectrum access strategies can be classified into three models, e.g., the dynamic exclusive use model, the open sharing model, and the hierarchical model. Among those models, the hierarchical model is a hierarchical access structure for primary users (PUs) and secondary users (SUs), and is the most promising and effective one for current spectrum access policies [1]. The basic idea of the hierarchical model is that the SUs can use the licensed spectrum of PUs, as long as they can limit any interference perceived by PUs. Furthermore, there are two models of the spectrum sharing between PUs and SUs, namely spectrum underlay and spectrum overlay.

Spectrum underlay introduces severe constraints on the transmission power of the SUs, therefore, it spreads the transmitted signals over a wide frequency band. The SUs can achieve low data rates with very low transmission power. If the PUs transmit in all the time-slots, the spectrum underlay does not need to detect and perceive the spectrum of the PUs.

Spectrum overlay, first presented by Mitola, can be also regarded as opportunistic spectrum access (OSA). Compared to the spectrum underlay, this model needs to detect and perceive the spectra of the PUs. It finds spatial and temporal spectrum white space for SUs to use, which is also termed as the spectrum holes (SHs). Therefore, this model does not need to obey the severe transmission power constraints of the SUs, and the SUs can achieve high date rates with high transmission power.

In most cases, the spectrum overlay and underlay models are used separately. In this paper, a hybrid spectrum access model is proposed to use both the overlay and underlay methods simultaneously to further improve the current spectrum efficiency.

The spectrum hole (SH) is a part of the licensed spectrum which is not being used by the owner during a period of time [1]. Among key technologies in CR, the design of the control channel is essential because the SUs need a control channel to coordinate and they have no licensed spectrum to carry the control information. The vulnerabilities resulting from utilizing a dedicated control channel have been well studied. Existing studies of the control channel have shown that using SHs to convey control information is only a basic approach and many shortcomings have been pointed out [2,3,4,5,6]. Firstly, the SUs may not have a common SH as control channel which would lead to low connectivity of the SUs. Secondly, the arrival of PU is unknown which causes interruptions in the use of the control channel.

As the SUs communicate only in the SHs, the SUs need information about those unused bands in which the PUs are inactive. Each SU should maintain a list of SHs which probably will differ from one to another. The SUs can communicate with each other if there is a common SH in their lists. Consequently, there should be a way to pass information about the lists between SUs during the initial communication.

Most of the existing MAC protocols of CR sensor networks are focused on avoiding common control channels. However, in this paper, a new method of spreading the power spectrum density in a control channel over an ultra-wide bandwidth is proposed to exploit the underused (gray) spectral regions. Like underlay spectrum sharing, the SUs can always access to the spectrum as long as the interference causing by SUs at the PU receiver can satisfactorily meet the threshold constraint [7].

According to the above analysis and considering the low power spectrum density of underlay waveforms, we propose to design a control channel to convey a small amount of control information, which is termed as SUCCH. At the same time, the spectrum overlay waveform is adopted to exchange a large amount of date, which is named as SUTCH. Our study is based on a spectrum sharing system consisting of two different waveforms. The first one is the Direct Sequence Code Division Multiple Access (DS-CDMA), which is defined as the underlay waveform used to convey control information. The second one is the Non-Contiguous Orthogonal Frequency Division Multiplexing (NC-OFDM), which is defined as the overlay waveform used to convey data information. The spectrum of NC-OFDM-based SUs is shared with the PUs which utilize DS-CDMA. Spreading Gain of DS-CDMA provides the required anti-jamming capability for the interference which may be caused by the SUs. In the meantime, based on the properties of the non-continuous power spectrum of NC-OFDM, it is more flexible for the SUs to access the SHs which are discontinuous in the frequency spectrum [8]. It is of great significance to discuss and study this issue, since the existing DS-CDMA is anticipated to be one of the spectrum sharing applications used in the future [9].

In order to set up the hybrid spectrum access model, several questions should be answered. The first one is the procedure for network setup between two SUs. The second one is the maximum access capacity of the SUTCH with different strategies. The third one is the reliability of the SUCCH. The fourth one is the power allocation strategies of the SUs between the SUTCH and SUCCH. In the rest of this paper, the above questions will be answered in detail. Specifically, Section 2 builds application scenarios and proposes a mechanism for establishing the CR sensor networks. In Section 3, a transmission channel model for analyzing the maximum access capacity for different polices with different objectives in the fading environment will be discussed. In Section 4, the reliability of the SUCCH is analyzed, and a hybrid spectrum access algorithm based on reinforcement learning model is proposed for the power allocation problem of the SUTCH and the SUCCH. Finally, Section 5 presents our simulation results and Section 6 concludes the paper.

## 2. Application Scenarios

**Figure 2:**
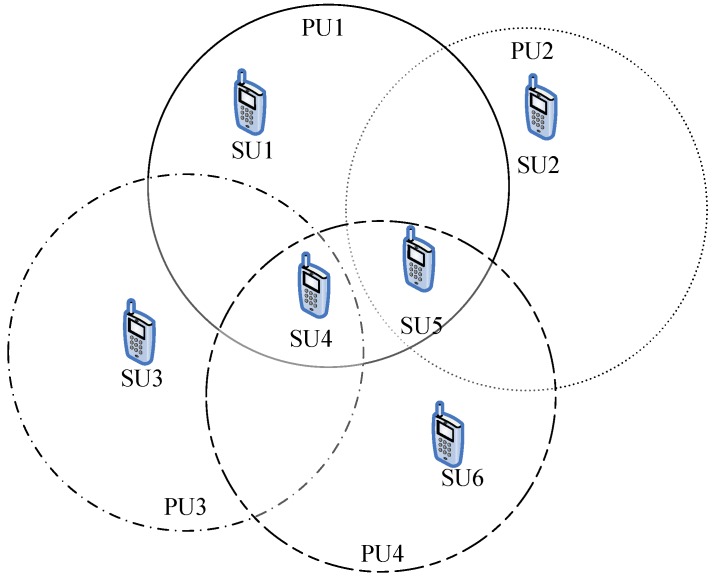
The SUs among four PUs.

In this section the application scenario is described as below. As shown in Figure 2, there are four active PUs and each one is authorized to use a certain frequency band to communicate. The different types of circles represent the interference ranges of each PU, and six SUs are shown in Figure 2. In this paper there is a channel which is termed a SH and a SU that can communicate in this channel because it is a channel whose authorized PU is currently inactive or the SU is beyond the interference range of that PU.

**Figure 3:**
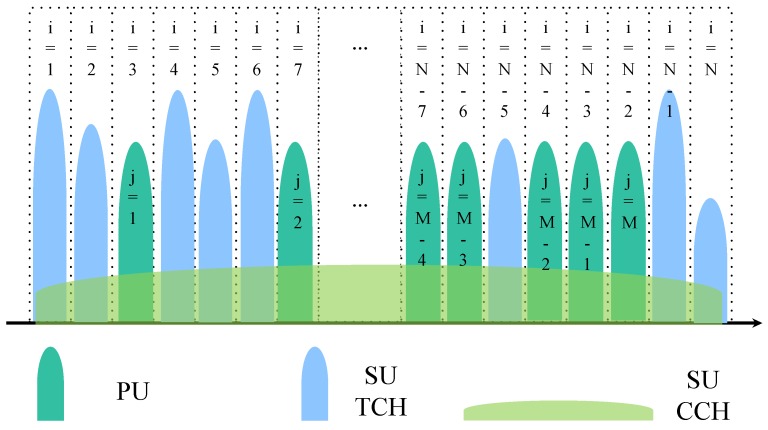
Spectral occupancy of the hybrid access.

A SU can establish the connection with another SU as long as they both have a shared SH in their respective lists of SHs, so it is important for a SU to identify its neighbors during the initial communication used to set up CR sensor networks. In order to fully utilize the primary spectrum and maximize the efficiency of spectrum, underlay and overlay transmissions, which exploit the white and grey spaces respectively, should be used together [1,10,11]. However, for spectrum underlay, the SUs need to transmit at low power to avoid any interference with the PUs, whereas the PUs will cause interference with SUs [12]. In consideration of the low power spectrum density of underlay waveforms, the control channel is designed to convey a small amount of control information, which is named as SUCCH, while the spectrum overlay waveform is used to exchange a large amount of data, which is named as SUTCH. Considering the perspective of a SU, the current spectrum usage is depicted in Figure 3.

Before explaining the protocol used to set up CR sensor networks, it is necessary to discuss the capabilities of the SUs and define some terms that will be used in the coming discussion. A SU can switch between spectra autonomously and sense the spectrum. Each SU identifies itself by using a different Orthogonal Variable Spreading Factor (OVSF) [12] over spectrum underlay. The number of the SUs in the current CR sensor networks is a priori information available to all the SUs.

**Figure 4:**
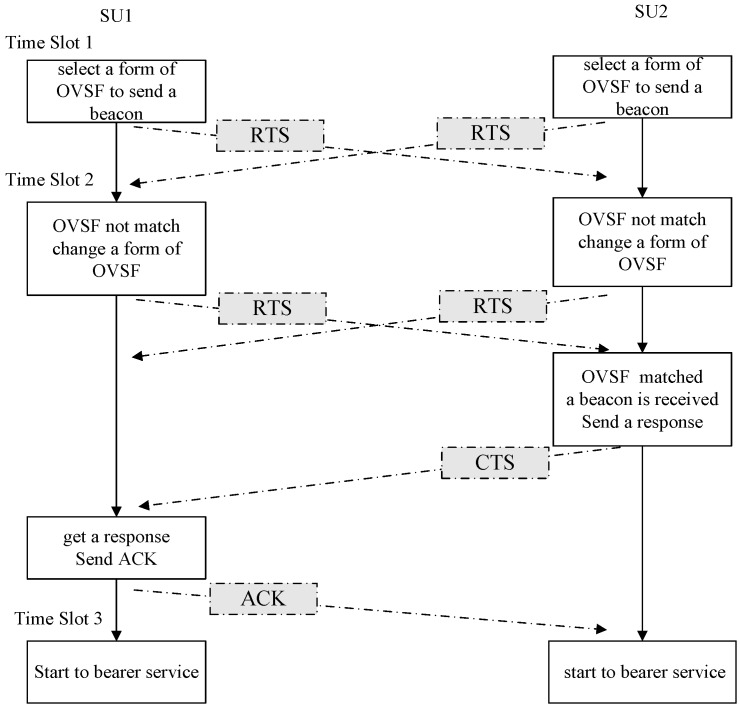
The procedure of network setup between two SUs.

The proposed protocol is firstly discussed under a distributed architecture scenario, which is also called Multi-Hop Architecture. Each SU initially starts to send beacons in different OVSF over spectrum underlay to indicate its presence. At the mean time every SU monitors the spectrum underlay by randomly selecting a form of OVSF while initially starting a timer which counts to *T_S_* seconds. If none of those beacons is captured during the *T_S_* seconds, the SU will change to another form of OVSF in the next time slot. If a beacon is received by selecting the current form of OVSF, the SUs will sent a response in the same form which is considered as the task of carrying on the negotiations. After exchanging the control information with each other, the common SH in the two SUs will start to provide service. The procedure is simply illustrated in Figure 4.

In Figure 4, “Request to Send (RTS)” and “Clear to Send (CTS)” exchange messages to reserve a channel for communications in a similar manner that the IEEE 802.11 Distributed Coordination function (DCF) designs the MAC protocol [13]. RTS or CTS carries information about SUs’ lists of SH and accesses SUs states.

## 3. Subchannel Selection Policies

Suppose the wireless channel is a frequency-selective Additive White Gaussian Noise (AWGN), the bandwidth is *B* Hz, and the power spectral density is *N*_0_. In this paper, it is divided into *N* Rayleigh fading subchannels, and the subchannel coherence bandwidth is ∆*f* Hz. Therefore, *B* = *N*∆*f*. These subchannels are indexed by *i* = 1, 2, …, *N*, and the gains of every subchannel are independent and identically distributed (i.i.d).

Active PUs use DS-CDMA technology to access the spectrum band with spreading gain *G*. According to the Central Limit Theorem, the interference process in the receiver of the SUs caused by a large number of PUs is considered a Gaussian approximation. Furthermore, according to the second-order statistics, the interference process is a white process [14]. Therefore, in each subchannel, the average interference introduced by the PUs at the receiver of the SUs is (*K* − 1)*N*_0_∆*f*, *K* ≥ 1, where *K* is a system parameter related to the characteristics of PUs network [15].

As shown in Figure 4, the SUs utilize NC-OFDM to access the SUTCH which is indexed by *j* = 1, 2, …, *M*, 0 ≤ *M* ≤ *N*. The SUs spread their SUCCH power spectrum density over an ultra-wide bandwidth to exploit the underused (gray) spectral. *Q* is defined as the interference threshold of the PUs, which is the maximum allowable temporal interference in the receiver of the PUs caused by concurrent activity of the SUs in the same subchannel. As mentioned in Figure 4, the protocol to set up CR sensor networks is based on the time-slot structure. Therefore, in order to satisfy the interference threshold constraint, the power of the SUs accessing the SUTCH should be controlled in each time-slot.

**Figure 5:**
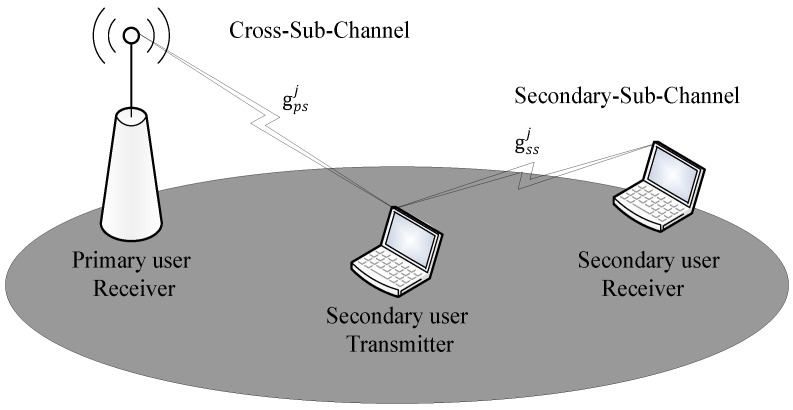
The structure of the accessing system for subchannel *j*.

In this paper, the structure of the accessing system is depicted in Figure 5. For subchannel *j*, the instantaneous gain between the transmitter and receiver of the SU is defined as $g_{ss}^{j}$, and the instantaneous gain between the transmitter of the SU and the receiver of the PU is defined as $g_{ps}^{j}$. Subscripts *s* and *p* refer to the secondary and the primary user, respectively. The $g_{ss}^{j}$ and $g_{ps}^{j}$ are assumed as the stationary and ergodic independent distributed random variables with unit-mean. Their Probability Density Functions (PDFs) are defined as $f_{ss}^{j}(g_{ss}^{j})$ and $f_{ps}^{j}(g_{ps}^{j})$, respectively. Channel gains $g_{ss}^{j}$ and $g_{ps}^{j}$ are i.i.d., *j* = 1, 2, …, *M*. In this paper, we suppose the perfect Channel Side Information (CSI) pair ($g_{ss}^{j},\;g_{ps}^{j})$ can be available in the transmitters. Here, the CSI contains the probability distribution of the channel gain, as well as the actual value at a certain time-slot. Actually, the CSI pair can be estimated by a spectral coordinator or proper signaling. Note that, the result derived from this assumption is an upper-bound in the case without a perfect CSI pair.

In this paper, we focus on the maximum achievable spectrum capacity of SUTCH, which is studied [16,17]. Since more than one SUs will compete to access to the underused frequency band. The SUs’ total available spectrum capacity is upper-bound by the case of only one SU, which is due to the fact that SUs will impose interference on each other. Therefore, the discussion of the individual SU can also be used as the upper-bound of the total spectrum capacity of all SUs.

At a given time-slot, the power allocation policy of SUTCH is defined as $\rho_{\psi }$, which is based on a selection criterion *ψ*(,..,), and set:

(1) $$\mu_{j}\overset{\Delta }{=}\psi \left(g_{ps}^{j},g_{ss}^{j}\right)$$

For the observing random variables *μ_j_*, *j* = 1, …, *M*, the selection sequence ***γ****_M_* is defined as follows:

(2) $$\gamma_{M}=\left(\mu_{r_{1}},\mu_{r_{2}},\ldots ,\mu_{r_{M}}\right)\overset{\Delta }{=}\rho_{\psi }\left(\mu_{1},\mu_{2},\ldots ,\mu_{M}\right)$$

The *M*-tuple selection sequence is arranged, so that its first element is the most suitable subchannel for SUTCH based on the selection criteria in Equation (1). The probability distribution function of random variable *γ_j_* is defined as *k_j_*(*γ*), *j* = 1, …, *M.* It is important to note that if *j*_1_, *j*_2_ are entities in ***γ****_M_* and *j*_1_ < *j*_2_, then it can be considered that compared to the choice *j*_2_, the SUs can get a better performance by choosing subchannel with index *j*_1_.

Suppose $\psi \left(g_{ps}^{j},g_{ss}^{j}\right)$ is constant, which means subchannels are considered equally. The SUs will randomly choose *M* out of *N* subchannels without any a priori information. This selection strategy is defined as the uniform subchannel selection, whereas, if the prior information of the subchannel obtained by cooperation or other techniques is −1, the SUs will choose the corresponding value of $\psi \left(g_{ps}^{j},g_{ss}^{j}\right)$. This selection strategy is defined as the non-uniform selection strategy.

The transmission power of the SUTCH in the subchannel *j* is referred to *P_sj_*. ***P****_s_*(*P_s_*_1_, …, *P_sM_*) is defined as the transmission power vector of SUTCH over *M* subchannels. Suppose that SUTCH accesses to the chosen subchannel *j* with the transmission power of *P_sj_*, and the corresponding interference at the receiver of the PUs is *Q_j_*, where:

(3) $$Q_{j}=g_{ps}^{j}P_{sj}$$

Since the PUs utilize DS-CDMA with spreading gain *G*, therefore, the narrow-band interference *Q_j_* spreads over the whole bandwidth and manifests itself as an equivalent wide-band interference equal to *G*^−1^*Q_j_* at the receiver of the PUs. Suppose the SUTCH transmits with the transmission power vector ***P****_s_*(*P_s_*_1_, *P_s_*_2_, …, *P_sM_*) in *M* accessible subchannels. Correspondingly, an equivalent narrow-band interference vector ***Q*** = (*Q*_1_, *Q*_2_, …, *Q_M_*) will be imposed on the receivers of the PUs. Meanwhile, the SUCCH transmits with the transmission power vector ***P****_sc_*(*P_sc_*_1_, *P_sc_*_2_, …, *P_scN_*). Therefore, in order to comply with the interference threshold *Q* of the PUs, the constraint function is as follows:

(4) $$\frac{1}{G}\left(\sum_{j=1}^{M}g_{\mathrm{ss}}^{j}P_{sj}+\sum_{i=1}^{N}g_{ps}^{i}P_{sci}\right)\leq Q$$

In this paper, the objective is to achieve the maximum capacity of SUTCH. As discussed above, the transmitting power of SUTCH in each accessible subchannel should be optimally allocated. Meantime, the interference threshold constraint should also be considered. Consequently, according to selection policy $\rho_{\psi }$, for a given *Q* and for *M* accessible subchannels, the maximum capacity of SUTCH is defined as $C_{M}^{\psi }$, which can be obtained by the following constrained optimization problem:

(5) $$\begin{array}{lll} C_{M}^{\psi } & = & \underset{P_{s}}{\max}\sum_{j=1}^{M}\Delta f\int_{g_{ps}^{j}g_{ss}^{j}}\log\left(1+\frac{g_{ss}^{j}P_{sj}}{KN_{0}\Delta f+g_{ss}^{j}P_{scj}}\right)\\ & & \times f_{ss}^{j}\left(g_{ss}^{j}\right)f_{ps}^{j}\left(g_{ps}^{j}\right)dg_{ss}^{j}dg_{ps}^{j}\\ s.t. & & \frac{1}{G}\left(\sum_{j=1}^{M}g_{ps}^{j}P_{sj}+\sum_{i=1}^{N}g_{ps}^{i}P_{sci}\right)\leq Q\\ & & \sum_{j=1}^{M}P_{sj}+\sum_{i=1}^{N}P_{sci}\leq P_{s} \end{array}$$

where, *Q* is the interference threshold of the PUs, which is the maximum allowable temporal interference in the receiver of the PUs caused by concurrent activity of the SUs in the same subchannel. *P_s_* *N*_0_ is the power spectral density, ∆*f* is the subchannel coherence bandwidth. *K* is a system parameter related to the characteristics of PUs network [15] within the range of 2–8. Equation (5) is derived from Shannon’s Capacity formula with the SUs power vector ***P****_s_* and *P_sc_*. Equation (6) is the constraint function of interference threshold of the PUs and maximum transmitting power of the SUs.

Actually, in contrast to the constraint of maximum transmitting power of the SUs, the constraint function of interference threshold of the PUs is much tighter [18]. Therefore, in this paper, the constraint of maximum transmitting power of SUs is not considered. At the same time, as mentioned above, the SUCCH spreads over an ultra-wide bandwidth to exploit the underused spectrum with a very low PSD, therefore, the interference caused by SUCCH is very low. In this paper, in order to simplify the analysis, the effect of SUCCH will not be considered, and Equation (5) will be further simplified as follows:

(6) $$\begin{array}{lll} C_{M}^{\psi } & = & \underset{P_{s}}{\max}\sum_{j=1}^{M}\Delta f\int_{g_{ps}^{j}g_{ss}^{j}}\log\left(1+\frac{g_{ss}^{j}P_{sj}}{KN_{0}\Delta f}\right)\\ & & \times f_{ss}^{j}\left(g_{ss}^{j}\right)f_{ps}^{j}\left(g_{ps}^{j}\right)dg_{ss}^{j}dg_{ps}^{j}\\ s.t. & & \frac{1}{G}\left(\sum_{j=1}^{M}g_{ps}^{j}P_{sj}\right)\leq Q \end{array}$$

Suppose $\psi \left(g_{ps}^{j},g_{ss}^{j}\right)$ = 1, thus the SUs will randomly choose *M* out of *N* subchannels without any priori information by *ρ*_1_, which is a uniform subchannel selection policy. Consequently, substituting Psj=Qjgpsj, j = 1,…,M and defining θQj≜QjKN0Δf Equation (6) can be simplified as follows:

(7) $$\begin{array}{lll} C_{M}^{\rho_{1}} & = & \underset{Q}{\max}\sum_{j=1}^{M}\Delta f\int_{\nu_{j}}\log\left(1+\nu_{j}\theta_{Q_{j}}\right)h_{j}\left(\nu_{j}\right)d\nu_{j}\\ s.t. & & \sum_{j=1}^{M}Q_{j}=GQ,\;\begin{array}{c} 0\leq Q_{j}\leq GQ \end{array} \end{array}$$

where vj≜gssjgpsj, 0 ≤ *v_j_* ≤ ∞, $v_{j}$ is the reward factor of the subchannel *j*. $\theta_{Q_{j}}$ is defined as the spectrum sharing load factor of the subchannel *j*.

Suppose the statistics characteristics of $\sqrt{g_{ps}^{j}},\sqrt{g_{ss}^{j}}$ is i.i.d. Rayleigh random variables, $g_{ps}^{j}$ and $g_{ss}^{j}$ are exponentially distributed random variables with unit-mean, therefore, the PDF of $v_{j}$ can be converted into [17]:

(8) $$\begin{array}{ll} h_{j}(\nu_{j}) & =\frac{d}{dv_{j}}\int_{0}^{\infty }\int_{0}^{g_{ps}^{j}v_{j}}e^{-g_{ps}^{j}}e^{-g_{ss}^{j}}dg_{ps}^{j}dg_{ss}^{j}\\ & =\int_{0}^{\infty }g_{ps}^{j}e^{-g_{ps}^{j}}e^{-g_{ps}^{j}\frac{g_{ss}^{j}}{g_{ps}^{j}}}dg_{ps}^{j}\\ & =\int_{0}^{\infty }g_{ps}^{j}e^{-g_{ps}^{j}\left(1+\nu_{j}\right)}dg_{ps}^{j}\\ & =-\frac{1}{1+\nu_{j}}\left\{{\left[g_{ps}^{j}e^{-g_{ps}^{j}\left(1+\nu_{j}\right)}\right]|}_{0}^{\infty }-\int_{0}^{\infty }e^{-g_{ps}^{j}\left(1+\nu_{j}\right)}dg_{ps}^{j}\right\}\\ & =-\frac{1}{{\left(1+\nu_{j}\right)}^{2}}{\left[e^{-g_{ps}^{j}\left(1+\nu_{j}\right)}\right]|}_{0}^{\infty }\\ & =\frac{1}{{\left(1+\nu_{j}\right)}^{2}}\;0<\nu_{j}<\infty \end{array}$$

Substituting Equation (9) into Equation (7), and integrating by part, Equation (10) can be gotten as follows, which is the simplified optimization problem of $C_{M}^{\rho_{1}}$:

(9) $$\begin{array}{ll} C_{M}^{\rho_{1}} & =\underset{\theta_{Q}}{\max}\sum_{j=1}^{M}\Delta f\frac{\theta_{Q_{j}}}{\theta_{Q_{j}}-1}\log\left(\theta_{Q_{j}}\right)\\ s.t. & \sum_{j=1}^{M}\theta_{Q_{j}}=GN\theta_{Q},\;0\leq \theta_{Q_{j}}\leq GN\theta_{Q} \end{array}$$

where, $\theta_{Q}$ is defined as the spectrum sharing load factor, and $\theta_{Q}=(\theta_{Q_{1}},\theta_{Q2},\ldots ,\theta_{QM})$ is defined as the spectrum sharing load vector:

(10) $$\theta_{Q}\overset{\Delta }{=}\frac{Q}{KN_{0}N\Delta f}=\frac{Q}{KN_{0}B}$$

Furthermore, the following pseudo linear approximation is used to get an approximate solution for Equation (10) [16]:

(11) $$\frac{x}{x-1}\log(x)\approx -1.2015-0.0052x+1.0772\times \log\left(3.0262x+308829\right)$$

Substituting Equation (12) into Equation (10), the Lagrangian function of the optimization problem Equation (10) is shown as follows [19,20]:

(12) $$\begin{array}{ll} L(\theta_{Q},\lambda )= & \sum_{j=1}^{M}-1.2015+-0.0052\times \theta_{Q_{j}}+1.0772\times \log\left(3.0262\times \theta_{Q_{j}}+3.8829\right)\\ & -\lambda \left(\sum_{j=1}^{M}\theta_{Q_{j}}-GN\theta_{Q}\right) \end{array}$$

where *λ* is the Lagrangian coefficient. The derivative with respect to the $\theta_{Q_{j}}$ on Equation (13) is taken, and then it is equal to zero, the following formula can be obtained:

(13) $$\theta_{Q_{j}}^{*}=\frac{1.0772}{\lambda^{*}+0.0052}-\frac{3.8829}{3.0262}.$$

Substituting Equation (14) into Equation (10), the following formula can obtained:

(14) $$\sum_{j=1}^{M}\left[\frac{1.0772}{\lambda^{*}+0.0052}-\frac{3.8829}{3.0262}\right]=GN\theta_{Q}$$

Equivalently, Equation (16) can be derived from Equation (15):

(15) $$\lambda^{*}=-0.0052+\frac{1.0772}{\frac{GN\theta_{Q}}{M}+\frac{3.8829}{3.0262}}.$$

Eventually, substituting Equation (16) into Equation (14) gives:

(16) $$\theta_{\theta_{j}}^{*}=\frac{GN\theta_{Q}}{M},\;j=1,2,\ldots ,M$$

Note that Equation (17) suggests that for given *G*, *N*, *M* and *θ_Q_*, the maximum capacity is achieved by dividing the total acceptable interference *GNθ_Q_* into equal portions for *M* accessible subchannels. Actually, it is a direct consequence of selecting *M* out of *N* subchannels without any prior knowledge. Furthermore, according to Equation (3) and θQj≜QjKN0Δf, the optimal transmitting power vector $P_{s}^{*}$ can be obtained as follows:

(17) $$P_{s}^{*}=\left(\frac{1}{g_{ps}^{1}}\frac{GQ}{M},\frac{1}{g_{ps}^{2}}\frac{GQ}{M},\ldots ,\frac{1}{g_{ps}^{M}}\frac{GQ}{M}\right)$$

Equation (18) suggests that the interference share for each accessible subchannel *j*, $\theta_{Q_{j}}$ is mapped to the corresponding transmission power *P_sj_*, proportional to 1/$g_{ps}^{j}$. So, if $g_{ps}^{j}$ is large, then the SUs will creates a large interference in the receivers of Pus. In this case, Equation (18) suggests a lower SUs transmission power in accessible subchannel *j*.

Equivalently, substituting Equation (18) into Equation (10), Equation (19) can be derived:

(18) $$C_{M}^{\rho_{1}}\approx M\Delta f\frac{GN\theta_{Q}}{GN\theta_{Q}-M}\log\left(\frac{GN\theta_{Q}}{M}\right)$$

In a practical case, *Q* = *G*^−1^*N*_0_*B* and *M* < *N*, the spectrum sharing load factor can be obtained from Equation (17) as $\theta_{\theta_{j}}=N/KM$, which is much higher than unity.

As mentioned above, $\rho_{1}$ randomly choose subchannels, which ignores the fact that it is more reasonable for the SUs to allocate higher transmission power to certain subchannels because of their corresponding CSIs, so it is essential to discuss the non-uniform selection policy for SUTCH with a prior knowledge of CSIs pair ($g_{ss}^{j}$, $g_{ps}^{j}$), since it will lead to a larger capacity or a smaller interference on the PUs.

Actually, an appropriate selection policy should consider the interference of the PUs receivers caused by SUs transmission. Such policy should select the lower subchannel gain of $g_{ps}^{j}$, because it will create a lower interference in the receivers of the PUs. Therefore, a lower $g_{ps}^{j}$ will give the SUs the flexibility of allocating a higher power, which will result in a higher capacity. Such a selection policy is named as SU-PU-based selection policy, which is simplified as $\rho_{ps}$. In order to implement $\rho_{ps}$, the SUs requires $g_{ps}^{j}$ during each time-slot. Therefore, a signaling channel between the receivers of the PUs and the transmitters of the SUs is required.

Similar to $\rho_{ps}$, another selection policy can be derived. It will select those subchannels which achieve the highest capacity corresponding to allocating the transmitting power of SUs. Such policy selects the subchannel with the higher $g_{ss}^{j}$, because it will create a higher power in the receivers of the SUs. Such selection is name as SU-SU-based selection policy, which is simplified as $\rho_{ss}$. In order to implement $\rho_{ss}$, the SUs requires $g_{ss}^{j}$ during each time-slot. Therefore, a signaling channel between the receivers of the SUs and the transmitters of the SUs is also required. In the following, the maximum capacity is derived with different selection policy $\rho_{ps}$ and $\rho_{ss}$.

Considering $\rho_{ps}$, the selection criteria can be assumed as follows:

(19) $$\psi \left(g_{ps}^{j},g_{ss}^{j}\right)=g_{ps}^{j}$$

Consequently, $\mu_{j}=g_{ps}^{j}$ and based on *μ_j_*, *j* = 1, 2, …, *M*, the selection sequence is defined as follows:

(20) $$\gamma_{M}=\left(\mu_{1},\mu_{2},\ldots ,\mu_{M}\right)\overset{\Delta }{=}\rho_{ps}\left(\mu_{1},\mu_{2},\ldots ,\mu_{M}\right)$$

where *μ*_1_ ≤ *μ*_1_ ≤ … ≤ *μ_M_*. Using order statistics [21], the probability distribution function of *μ_j_*, ∀*j* is shown as follows:

(21) $$k_{j}(\mu )=N_{j}F_{\mu }^{j-1}\left(\mu \right){\left[1-F_{\mu }\left(\mu \right)\right]}^{N-j}f_{\mu }\left(\mu \right)$$

where:

(22) $$N_{j}\overset{\Delta }{=}\frac{N!}{(j-1)!(N-j)!},$$

and *f_μ_*(*μ*), *F_μ_*(*μ*) are the probability density function and probability distribution function of *μ*. Assuming the same assumption as discussed above in Equation (9) we obtain:

(23) $$f_{\mu }(\mu )=e^{-\mu },F_{\mu }(\mu )=1-e^{-\mu }.$$

Equivalently:

(24) $$k_{j}(\mu )=N_{j}{\left(1-e^{-\mu }\right)}^{j-1}e^{-\mu (N-j+1)}.$$

Using a binomial expansion to replace (1 − *e*^−*μ*^)*^j^*^−1^ in Equation (25) gives:

(25) $$k_{J}(\mu )=N_{j}\sum_{l=0}^{j-1}F_{l}^{j-1}e^{-\mu (N-l)},$$

where, $F_{l}^{j-1}≜\left(\begin{array}{c} j-1\\ l \end{array}\right){\left(-1\right)}^{j-1-l}$.

Thus, the optimization problem of maximizing the capacity of SUTCH, while satisfying the tolerable interference constraint of the PUs with selection policy $\rho_{ps}$ is shown as follows:

(26) $$\begin{array}{lll} C_{M}^{\rho_{ps}} & = & \underset{\theta_{Q}}{\max}\sum_{j=1}^{M}\sum_{l=0}^{j-1}\Delta fN_{j}F_{l}^{j-1}\frac{\theta_{Q_{j}}\log\left[\left(N-l\right)\theta_{Q_{j}}\right]}{\left(N-l\right)\theta_{Q_{j}}-1},\\ s.t. & & \sum_{j=1}^{M}\theta_{Q_{j}}=GN\theta_{Q_{j},}\;0\leq \theta_{Q_{j}}\leq GN\theta_{Q}. \end{array}$$

However, in practice, *M* < *N*, thus, $N\theta_{Q_{j}}\gg 1$. Therefore, Equation (27) can be approximated by Equation (28):

(27) $$\begin{array}{lll} C_{M}^{\rho_{ps}} & \approx & \underset{\theta_{Q}}{\max}\sum_{j=1}^{M}\sum_{l=0}^{j-1}\Delta f\frac{N_{j}F_{l}^{j-1}}{N-l}\log\left[\left(N-l\right)\theta_{Q_{j}}\right],\\ s.t. & & \sum_{j=1}^{M}\theta_{Q_{j}}=GN\theta_{Q_{j},}\;0\leq \theta_{Q_{j}}\leq GN\theta_{Q}. \end{array}$$

The Lagrange multiplier algorithm can be used to solve the optimization problem in Equation (28) [19]:

(28) $$\begin{array}{ll} L(\theta_{Q_{j}},\lambda ) & =\sum_{j=1}^{M}\sum_{l=0}^{j-1}\frac{N_{j}F_{l}^{j-1}}{N-l}\log\left[\left(N-l\right)\theta_{Q_{j}}\right]\\ & -\lambda \left(\sum_{j=1}^{M}\theta_{Q_{j}}-GN\theta_{Q}\right) \end{array}$$

where, *λ* is the Lagrangian coefficient.

Taking the derivative with respect to the $\theta_{Q_{j}}$ on Equation (29) and setting it equal to zero gives:

(29) $$\theta_{Q_{j}}^{*}=\frac{1}{\lambda^{*}}\upsilon_{j},$$

where, vj≜∑l−0j−1NjFlj−1N−l. Substituting Equation (30) into Equation (28):

(30) $$\lambda^{*}=\frac{1}{GN\theta_{Q}}\sum_{j=1}^{M}\upsilon_{j}.$$

Substituting Equation (31) into Equation (30):

(31) $$\theta_{Q_{j}}^{*}=GN\theta_{Q}\frac{\upsilon_{j}}{\sum_{j=1}^{M}\upsilon_{j}}.$$

Furthermore, according to Equation (3) and θQj≜QjKN0Δf, the optimal transmitting power vector $P_{s}^{*}$ with selection policy $\rho_{ps}$ can be obtained as follows:

(32) $$P_{s}^{*}=\frac{GQ}{\sum_{j=1}^{M}\upsilon_{j}}\left(\frac{\upsilon_{1}}{g_{ps}^{j}},\frac{\upsilon_{2}}{g_{ps}^{j}},\ldots ,\frac{\upsilon_{M}}{g_{ps}^{j}}\right)$$

Equivalently, substituting Equation (33) into Equation (28) yields the approximated maximum achievable capacity of the SUTCH with selection policy $\rho_{ps}$, which is shown in Equation (34):

(33) $$C_{M}^{\rho_{ps}}\approx \sum_{j=1}^{M}\sum_{l=0}^{j-1}\frac{\Delta fN_{j}F_{l}^{j-1}}{N-l}\log\left[\left(N-l\right)GN\theta_{Q}\frac{\upsilon_{j}}{\sum_{j=1}^{M}\upsilon_{j}}\right]$$

Considering $\rho_{ss}$, the selection criteria can be assumed as follows:

(34) $$\psi \left(g_{ps}^{j},g_{ss}^{j}\right)=g_{ss}^{j}$$

Consequently, $\mu_{j}=g_{ss}^{j}$ and based on *μ_j_*, *j* = 1, 2, …, *M*, the selection sequence is defined as follows:

(35) $$\gamma_{M}=(\mu_{1},\mu_{2},\ldots ,\mu_{m})\overset{\Delta }{=}\rho_{ss}(\mu_{1},\mu_{2},\ldots \mu_{m}).$$

where *μ*_1_ ≥ *μ*_2_ ≥ … ≥ *μ_M_*. Using order statistics [21], the probability distribution function of *μ_j_*, ∀*j* is shown as follows:

(36) $$k_{j}(\mu )=N_{j}F_{\mu }^{N-j}(\mu ){\left[1-F_{\mu }(\mu )\right]}^{j-1}f_{\mu }(\mu ),$$

Using a binomial expansion to replace (1 − *e*^−*μ*^)*^N−j^* in Equation (37) one obtains:

(37) $$k_{J}(\mu )=N_{j}\sum_{l=0}^{N-j}F_{l}^{N-j}e^{-\mu (l+j)},$$

where $F_{l}^{N-j}≜\left(\begin{array}{c} N-j\\ l \end{array}\right){\left(-1\right)}^{l}$.

Thus the optimization problem of maximizing the capacity of the SUTCH while satisfying the tolerable interference constraints of the PUs with selection policy $\rho_{ps}$ is shown as follows:

(38) $$\begin{array}{lll} C_{M}^{\rho_{ss}} & = & \underset{\theta_{Q}}{\max}\sum_{j=1}^{M}\sum_{l=0}^{N-j}\Delta f\frac{N_{j}F_{l}^{N-j}}{l+j}\frac{\frac{\theta_{Q_{j}}}{l+j}}{\frac{\theta_{Q_{j}}}{l+j}-1}\log\left(\frac{\theta_{Q_{j}}}{l+j}\right)\\ s.t. & & \sum_{j=1}^{M}\theta_{Q_{j}}=GN\theta_{Q_{j}},\;0\leq \theta_{Q_{j}}\leq GN\theta_{Q}. \end{array}$$

Utilizing the following approximation for small values of θQjl+j, *l* = 0, 1, …, *N − j* as:

(39) $$\theta_{Q_{j}}^{*}=GN\theta_{Q}\frac{\chi_{j}^{2}}{\sum_{j=1}^{M}\chi_{j}^{2}}$$

where:

(40) $$\chi_{j}\overset{\Delta }{=}\sum_{l=0}^{N-j}N_{j}\frac{F_{l}^{N-j}}{2{\left(l+j\right)}^{3/2}}$$

Furthermore, according to Equation (3) and θQj≜QjKN0Δf, the optimal transmitting power vector $P_{s}^{*}$ with selection policy $\rho_{ss}$ can be achieved as follows:

(41) $$P_{s}^{*}=\frac{GQ}{\sum_{j=1}^{M}\chi_{j}^{2}}\left(\frac{\chi_{1}^{2}}{g_{ss}^{j}},\frac{\chi_{2}^{2}}{g_{ss}^{j}},\ldots ,\frac{\chi_{M}^{2}}{g_{ss}^{j}}\right)$$

Equivalently, substituting (41) into (39) yields the approximated maximum achievable capacity of the SUTCH under SU-SU-based selection policy is shown as follows:

(42) $$C_{M}^{\rho_{ss}}\approx \sum_{j=1}^{M}\sum_{l=0}^{N-j}\Delta f\frac{N_{j}F_{l}^{N-j}}{{\left(l+j\right)}^{3/2}}{\left(GN\theta_{Q}\frac{\chi_{j}^{2}}{\sum_{j=1}^{M}\chi_{j}^{2}}\right)}^{1/2}$$

## 4. Reinforcement Learning for Improving Performance

In Section 3, the maximum achievable capacity of the SUTCH is analyzed. In Section 4, the reliability of the SUCCH is taken into consideration by the Bit Error Rate (BER). Suppose the signal waveform of the SUCCH is as follows:

(43) $$\left\{ \begin{array}{l} s_{1}(t)=\sqrt{\varepsilon_{b}}\\ s_{2}(t)=-\sqrt{\varepsilon_{b}} \end{array}\right.$$

Suppose the two signal waveforms in Equation (45) are transmitted with the same probability. Since the SUCCH spreads its power spectrum density over an ultra-wide bandwidth to exploit the underused (gray) spectral regions, the interference process caused by the PUs and the SUCCH can be considered as a Gaussian approximation. If the SUCCH transmits *s_1_*(*t*), after the despread-demodulation algorithm at the receiver of the SUCCH, the received signal is as follows:

(44) $$r=\sqrt{\varepsilon_{b}}+\frac{1}{G_{\mathrm{SUCCH}}}\left(n+\sum_{y=1}^{Y}\sigma_{PU}+\sum_{j=1}^{M}\sigma_{\mathrm{SUCCH}}\right)$$

where *n* is additive Gaussian white noise with mean zero, variance *N*_0_/2 and *σ_PU_*, *σ*_SUCCH_ represent the interference caused by the PUs and the SUTCH. *G*_SUCCH_ is the spreading gain of the SUCCH. The receiving signal of the SUCCH is compared with the threshold zero, which is as follows:

(45) $$r\overset{\overset{S_{1}}{\geq }}{\underset{s_{2}}{<}}0.$$

Suppose the PUs and the SUCCH are i.i.d. random processes, then two probability density functions of *r* are given as follows:

(46) $$\begin{array}{l} p\left(r|s_{1}\right)=\frac{1}{\sqrt{\frac{2\pi }{G_{\mathrm{SUCCH}}}\left(\frac{N_{0}}{2}+\sum_{y=1}^{Y}\sigma_{pu}^{2}+\sum_{j=1}^{M}\sigma_{\mathrm{SUTCH}}^{2}\right)}}e^{-{\left(r-\sqrt{\varepsilon_{b}}\right)}^{2}/N_{0}}\\ p\left(r|s_{2}\right)=\frac{1}{\sqrt{\frac{2\pi }{G_{\mathrm{SUCCH}}}\left(\frac{N_{0}}{2}+\sum_{y=1}^{Y}\sigma_{pu}^{2}+\sum_{j=1}^{M}\sigma_{\mathrm{SUTCH}}^{2}\right)}}e^{-{\left(r+\sqrt{\varepsilon_{b}}\right)}^{2}/N_{0}} \end{array}$$

Consequently, the average error probability of the SUCCH is as follows:

(47) $$\begin{array}{ll} P_{e} & =\frac{1}{2}\left[P\left(e|s_{1}\right)+P\left(e|s_{2}\right)\right]\\ & =\frac{1}{2}\left[\int_{-\infty }^{0}p\left(r|s_{1}\right)dr+\int_{0}^{+\infty }p\left(r|s_{2}\right)dr\right]\\ & =Q\left(\sqrt{\frac{G_{\mathrm{SUCCH}}\varepsilon_{b}}{\frac{N_{0}}{2}+\sum_{y=1}^{Y}\sigma_{PU}^{2}+\sum_{j=1}^{M}\sigma_{\mathrm{SUCCH}}^{2}}}\right) \end{array}$$

Suppose the control information of the SUCCH consists of 8 bits. According to Figure 4, the transmitter and receiver of the SUs need to coordinate access to the spectrum three times. Therefore, the probability of successful establishment for the SUCCH can be concluded. Furthermore, the total interference caused by the SUs is divided into two parts: *Q*_SUTCH_ and *Q*_SUCCH_. *Q*_SUTCH_ represents the interference caused by the activity of the SUTCH, while *Q*_SUCCH_ represents the interference caused by the activity of the SUTCH. The loading factor Г is defined as the radio of *Q*_SUTCH_ and *Q*_SUCCH_, which is as follows:

(48) $$\Gamma \overset{\Delta }{=}\frac{Q_{\mathrm{SUCCH}}}{Q_{\mathrm{SUTCH}}},\;0<\Gamma <1$$

In consideration of the link access protocol design described above and the probability of successful establishment for SUCCH, the lower PSD of SUCCH means it may take more time to complete the setup procedure for the SUs. In other words, accessible subchannels will remain idle for a long period of time, which will lead to spectrum resource waste. However, increasing the transmitting power of the SUCCH will decrease the transmitting power of the SUTCH, because of the total interference constraint caused by the SUs is certain at a time-slot. Lower transmitting power of the SUTCH will lead to reduce the capacity of data. Therefore, it’s a trade-off, which is essential to choose the appropriate transmitting power of SUTCH according to the characteristic of the activity of the PU. For this purpose, a hybrid access method based on Reinforcement Learning model is proposed to solve this problem. The most prominent feature of Reinforcement Learning model is its autonomous learning and online learning ability. By trial and error, Reinforcement Learning model can get a better strategy based on the subchannel environment.

The Cross model [22] is now widely recognized as one of the Reinforcement Learning models with memory-less characteristics, which means the learning process is a Markov Decision Process (MDP). The basic idea is to follow the rules of “Results” [23], namely, if system is rewarded by choosing a strategy, then the next period will get higher probability of choosing such strategy. On the contrary, if it is punished, the next period will reduce the probability of choosing such strategy.

Bush and Mosteller [24] introduced the Bush-Mosteller model in 1955 [25]. Afterwards, Roth and Erev improved this model and introduced the Roth-Ever model. Nowadays, as two models of reinforcement learning, both of them [26] are widely adopted. They are easy to realize and have very low computation complexity, which fit for the real-time applications. Therefore, in this paper, these two models are introduced and some necessary modifications are adopted for the application, so the model of MDP Cross and Statistical Mean are proposed.

As mentioned above, the process of connection setup is defined as the time-slotted. The optional strategies for the SUs are defined as follows:

(49) $$A_{su}=\left(\Gamma_{1},\Gamma_{2},\ldots ,\Gamma_{n},\ldots ,\Gamma_{n^{\prime }},\Gamma_{R}\right)$$

where **A***_su_* is the vector of optional strategies, *R* the number of the strategy, *n* is the chosen strategy and *n*′ are not chosen strategies in a certain time-slot.

Consequently, during the time-slot *k* to access the initial stage, the SUs can update the probability of choosing strategy *n* and *n*′ by the following formula:

(50) $$\begin{array}{ll} p_{}^{n}\left(k+1\right)=p_{}^{n}\left(k\right)+R\left[u\left(k\right)\right]\times \left(1-p_{}^{n}\right) & n=A_{su}\left(k\right)\\ p^{n^{\prime }}\left(k+1\right)=p^{n^{\prime }}\left(k\right)-R\left[u\left(k\right)\right]\times p^{n^{\prime }}\left(k\right) & \;n^{\prime }\neq A_{su}\left(k\right)\\ R\left[u\left(k\right)\right]=\alpha \times u\left(k\right)+\beta & \end{array}$$

where *A_su_*(*k*) is the accessible strategy of the SUs at the time-slot *k*, which can be seen the action of MDP. *p^n^*(*k*) is the probability of the accessible strategy *n* of the SUs at time-slot *k*, *p^n^*(*k*) is the probability of the unused strategy *n*′ of the SUs at time-slot *k*, which can be seen as the state of MDP. *u*(*k*) is the reward function of the accessible performance of the SUs, which can be seen as the reward of MDP. *α* and *β* are the adjustment factors, which can be used to determine the updating rate of *u*(*k*). *R*[*u*(*k*)] is defined as the monotone function of *u*(*k*), which is −1 < *R*[*u*(*k*)] < 1. When the SUTCH successfully accesses idle subchannels, it obtains the reward, which is defined as follows:

(51) $$\partial_{1}I\left(k\right)C_{\mathrm{SUTCH}}\left(k\right)T\left(k\right)$$

where, *T*(*k*) is the transmission duration of the SUs in time-slot *k* and ∂_1_ is a weighting factor and *I*(*k*) is indicator function, which is defined as follows:

(52) $$\left\{ \begin{array}{ll} I\left(k\right)=1 & \mathrm{SUTCH}\;\mathrm{successfully}\;\mathrm{access}\;\mathrm{at}\;\mathrm{time-slot}\;k\\ I\left(k\right)=0 & \mathrm{SUTCH}\;\mathrm{fail}\;\mathrm{to}\;\mathrm{access}\;\mathrm{at}\;\mathrm{time-slot}\;k \end{array}\right.$$

When the SUTCH fails to access the idle subchannels, it wastes the opportunity for transmission and pays the cost, which is shown as follows:

(53) $$-\partial_{2}I\left(k\right)C_{\mathrm{SUTCH}}\left(k\right)T^{\prime }\left(k\right)$$

where, *T′*(*k*) is the access duration of the SUs and ∂_2_ is also a weighting factor.

Equivalently:

(54) $$u\left(k\right)=\partial_{1}I\left(k\right)C_{\mathrm{SUTCH}}\left(k\right)T\left(k\right)-\partial_{2}I^{\prime }\left(k\right)C_{\mathrm{SUTCH}}\left(k\right)T_{}^{\prime }\left(k\right)\;0\leq \partial_{i}\leq 1,i=1,2$$

In order to weaken the impact of weighting on updating the probability of the choosing strategy, Equation (52) can be further defined as follows:

(55) $$\begin{array}{ll} p_{}^{n}\left(k+1\right)=p_{}^{n}\left(k\right)+\varepsilon \times \left[1-p_{}^{n}\left(k\right)\right] & n=A_{su}\left(k\right),u\left(k\right)>0\\ p_{}^{n}\left(k+1\right)=p_{}^{n}\left(k\right)-\varepsilon \times p_{}^{n}\left(k\right) & n=A_{su}\left(k\right),u\left(k\right)<0\\ p^{n^{\prime }}\left(k+1\right)=p^{n^{\prime }}\left(k\right)+\varepsilon \times \left[1-p^{n^{\prime }}\left(k\right)\right] & \;n^{\prime }\neq A_{su}\left(k\right),u\left(k\right)<0\\ p^{n^{\prime }}\left(k+1\right)=p^{n^{\prime }}\left(k\right)-\varepsilon \times p^{n^{\prime }}\left(k\right) & n^{\prime }\neq A_{su}\left(k\right),u\left(k\right)>0 \end{array}$$

where, *ε* = *R*[*u*(*k*)] = *α* × *u*(*k*)+ *β*. The solution to update the probability of choosing strategy is the model of MDP Cross. If the *u*(*k*) > 0, which means the accessible strategy *n* is fit for the current subchannel environment. Therefore, the *p^n^*(*k* + 1) should be increased, while the *p^n^*^′^ (*k* + 1) should be decreased. However, if the *u*(*k*) < 0, which means the accessible strategy *n* is not fit for the current subchannel environment, therefore, the *p^n^*(*k* + 1) should be decreased, while the *p^n^*^′^(*k* + 1) should be increased.

In practice, the probability of choosing a strategy is usually not only dependent on the latest result, it also takes the “system history” into account. “System history” presents users with more information about the status of environment. In order to incorporate the “system history”, the Statistical Mean is proposed, in which the reward function is modified as follows:

(56) $$\begin{array}{l} p_{suc}^{n}\left(k\right)=F_{suc}^{n}\left(k\right)/F_{access}^{n}\left(k\right)\\ p_{fail}^{n}\left(k\right)=F_{fail}^{n}\left(k\right)/F_{access}^{n}\left(k\right)\\ u\left(k\right)=\partial_{1}p_{suc}^{n}\left(k\right)-\partial_{2}p_{fail}^{n}\left(k\right) \end{array}$$

where, $F_{suc}^{n}\left(k\right)$ represents the amount of data traffic which SUTCH has transmitted based on strategy *n* at time-slot *k*, $F_{access}^{n}\left(k\right)$ and $F_{fail}^{n}\left(k\right)$ are the idea and wasted amount, respectively.

Therefore the probability of choosing a strategy in the Statistical Mean is shown as follows:

(57) $$\begin{array}{ll} p_{}^{n}\left(k+1\right)=p_{}^{n}\left(k\right)+\varepsilon \times \left[1-p_{}^{n}\left(k\right)\right] & n=A_{su}(k),\forall j,j\neq n,u^{n}\left(k+1\right)>u^{j}\left(k+1\right)\\ p_{}^{n}\left(k+1\right)=p_{}^{n}\left(k\right)-\varepsilon \times p_{}^{n}\left(k\right) & n=A_{su}(k),\exists j,j\neq n,u^{n}\left(k+1\right)\leq u^{j}\left(k+1\right)\\ p^{n^{\prime }}\left(k+1\right)=p^{n^{\prime }}\left(k\right)+\varepsilon \times \left[1-p^{n^{\prime }}\left(k\right)\right] & \;n^{\prime }\neq A_{su}(k),u^{n}\left(k+1\right)\leq u^{n^{\prime }}\left(k+1\right)\\ p^{n^{\prime }}\left(k+1\right)=p^{n^{\prime }}\left(k\right)-\varepsilon \times p^{n^{\prime }}\left(k\right)) & n^{\prime }\neq A_{su}(k),u^{n}\left(k+1\right)>u^{n^{\prime }}\left(k+1\right) \end{array}$$

## 5. Simulation Study

In this section, the achievable spectrum efficiencies with different subchannel selection policies are compared. Here, the spectrum sharing load factor is *θ_Q_* = −30 dB and the number of subchannels is *N* = 40. The mean values of random variables $g_{ps}^{j},g_{ss}^{j}$ are denoted by $\lambda_{ps},\lambda_{ss}$, respectively. The achieved spectrum efficiency is defined as follows:

(58) $$C_{\rho_{\psi }}=C_{M}^{\rho_{\psi }}/M\Delta f$$

Here, in order to facilitate the comparison, $C_{\rho_{1}}$ is defined as the achieved spectrum efficiency with uniform subchannel selection, $C_{\rho_{ss}}$ is defined as the achieved spectrum efficiency with the SU-SU-based selection policy, $C_{\rho_{ps}}$ is defined as the achieved spectrum efficiency with the SU-PU-based selection policy.

**Figure 6:**
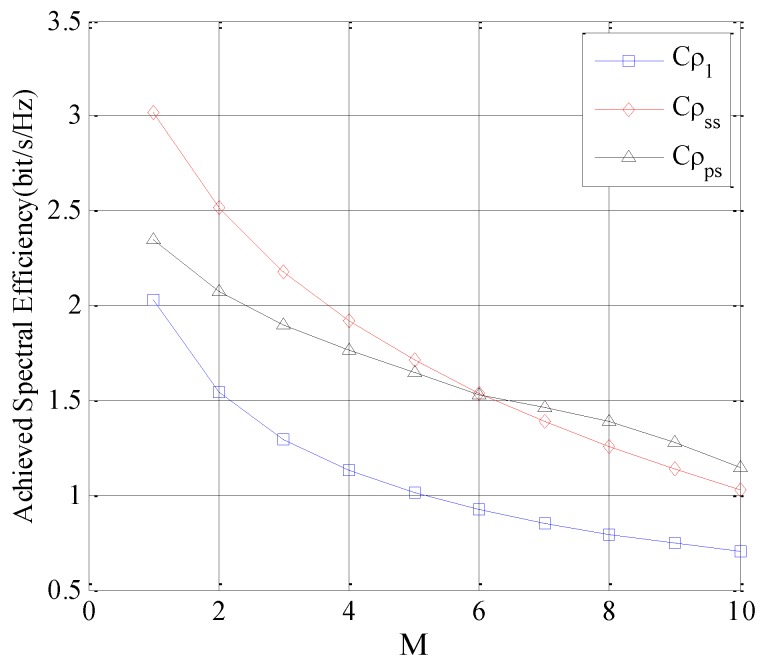
Achieved spectral efficiency of the SUTCH with three selection policies with *M*.

In the first simulation, suppose the interference threshold is a constant and $\lambda_{ps}=\lambda_{ss}$, and the $C_{\rho_{\psi }}$ is analyzed by increasing *M*, which is depicted in Figure 6.

As depicted in Figure 6, $C_{\rho_{1}}$ is lower than that of $C_{\rho_{ss}}$ and $C_{\rho_{ps}}$, therefore, it indicates that *ρ*_1_ has a poorer performance compared to *ρ_ss_* and *ρ_ps_*. For *M* = 1, the gap between $C_{\rho_{1}}$ and $C_{\rho_{ss}}$ is large. However, with the increase of *M*, the gap is reduced. This result is reasonable because the tap is related to the *M*/*N* ratio, and the larger *M*/*N*, the lower the tap is. The reason is that for a larger *M*/*N*, the set of *M* subchannels accessible by $C_{\rho_{1}}$ and $C_{\rho_{ss}}$ probably has a large overlap.

With the increase of *M*, the rate of decrease of *ρ_ps_* is reduced with the slowest rate. This is mainly due to the fact that the total interference threshold of the receivers of the PUs is a constant. At the same time, *ρ_ps_* selects these subchannels with the lower $g_{ps}^{j}$, which enables the SUs transmitters to send the maximum transmitting power, without generating high interference on the receivers of the Pus and satisfying the constraint of the interference threshold of the PUs. According to Figure 6, for a large number of accessible subchannels with constant interference constraint, *ρ_ps_* achieves a better performance.

**Figure 7:**
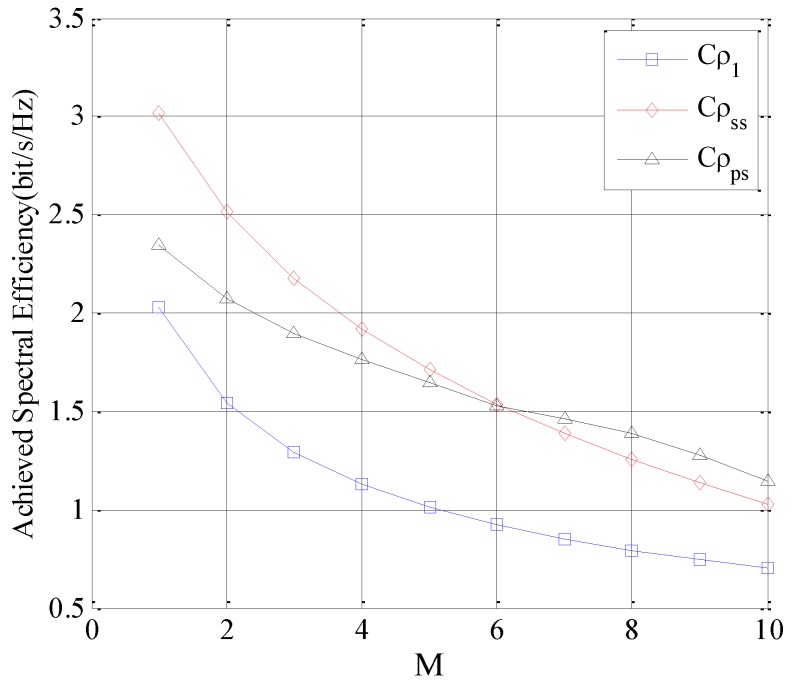
Achieved spectral efficiency of the SUTCH under three selection policies with *N*.

In the second simulation, the influence of the number of subchannels *N* is analyzed. Suppose *M* = 1, $\lambda_{ps}=\lambda_{ss}$, the $C_{\rho_{\psi }}$ is analyzed by increasing *N*. The result is depicted in Figure 7.

As seen in Figure 7, for all the different subchannel selection policies, the $C_{\rho_{\psi }}$ increases with the increase of *N*. This is because that the probability of selecting proper subchannels for SUTCH is increasing with *N*. Furthermore, it is interesting to find that the gap between these three selection policies also increases with the increase of *N* and *ρ_ss_* outperforms the others in this simulation.

**Figure 8:**
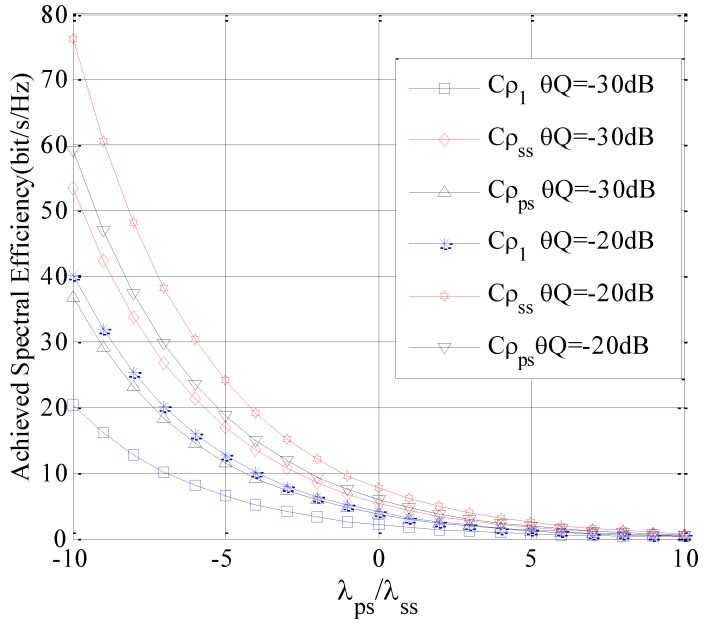
Achieved spectral efficiency of the SUTCH under three selection policies with $\lambda_{ps}/\lambda_{ss}$.

In the third simulation, both the influences of *g_ps_* and *g_ss_* are evaluated. Suppose *N* = 40, *M* = 1. The $C_{\rho_{\psi }}$ is analyzed with $\lambda_{ps}/\lambda_{ss}$ for different *θ_Q_* values. The simulation result is depicted in Figure 8.

As depicted in Figure 8, it is clearly observed that the $C_{\rho_{\psi }}$ of the SUTCH decreases with the increase of $\lambda_{ps}/\lambda_{ss}$. Meantime, the $C_{\rho_{\psi }}$ of the SUTCH decreases with the decrease of *θ_Q_*. This is due to the fact that with the increase of $\lambda_{ps}/\lambda_{ss}$, the attenuation of *g_ps_* is decreased while that of *g_ss_* is increased. Consequently, the $C_{\rho_{\psi }}$ of SUTCH is lower with the same transmitting power. On the other hand, with the decrease of *θ_Q_*, the power allocated to each selected subchannel is bound to be reduced, which will lead to the deterioration in the $C_{\rho_{\psi }}$ of the SUTCH.

Compared comprehensively, the $C_{\rho_{\psi }}$ of the SUTCH with *ρ*_1_ has the lowest value, since it just ignores any a priori knowledge of subchannel’s status. However, under different conditions, the performance of the *ρ_ss_* and *ρ_ps_* are different. When the ratio of *M*/*N* is small, the best subchannel selection policy is *ρ_ss_*. However, if the ratio of *M*/*N* is large, the best subchannel selection policy is *ρ_ps_*.

In the fourth simulation, as mentioned above, in Equation (49), the BER of SUCCH is derived. Therefore, Monte Carlo Simulation is used to prove its rationality. The simulation parameters are shown in Table 1. Suppose $\sigma_{PU}^{2}=\sigma_{\mathrm{SUTCH}}^{2}$.

**Figure 9:**
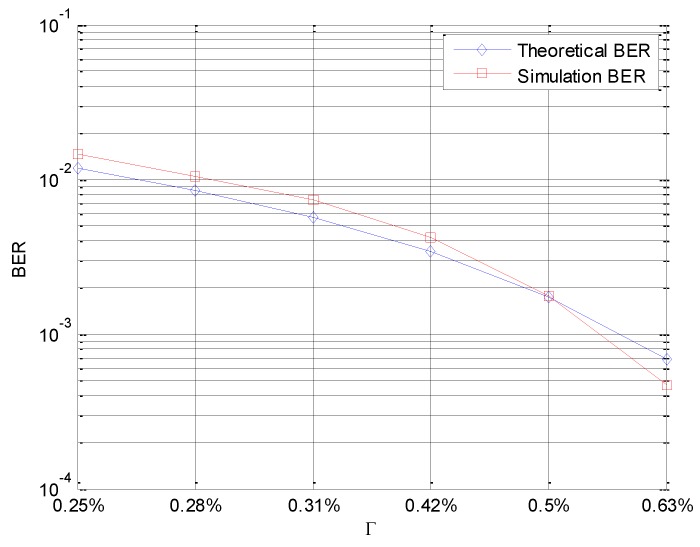
Theoretical and simulation BER versus different Г.

In Figure 9, the Simulation BER is calculated by Monte Carlo Simulation Experiment, while the Theoretical BER is calculated by Equation (49). As depicted in Figure 9, the simulation BER follows the Theoretical BER very closely.

As mentioned in Section 4, the trade-off problem between the reliability of the SUCCH and the efficiency of the SUTCH is discussed. Here, suppose the arrival rate of the authorized PUs accessing to the subchannels follows a Poisson distribution. Simulation parameters are shown in Table 2. Suppose $\lambda_{m}^{j}$ represents the arrival rate of the PUs in accessible subchannels.

In the fifth simulation, the achieved spectral efficiency, achieved data traffic and unused data traffic are used to compare the accessible performance of the three different selection policies. Here, achieved spectral efficiency represents the proportion between data traffic and unused data traffic. Data traffic is the total amount of unit data traffic when the SUTCH has successfully accessed to the idle subchannel, while unused data traffic is the achievable amount of unit data traffic during the time cost in establishing the connection.

**Figure 10:**
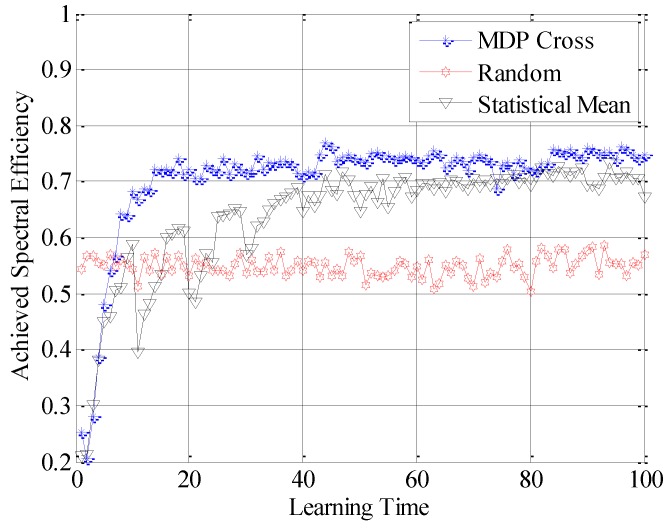
Achieved spectral efficiency of the SUTCH under three strategies with learning time.

**Figure 11:**
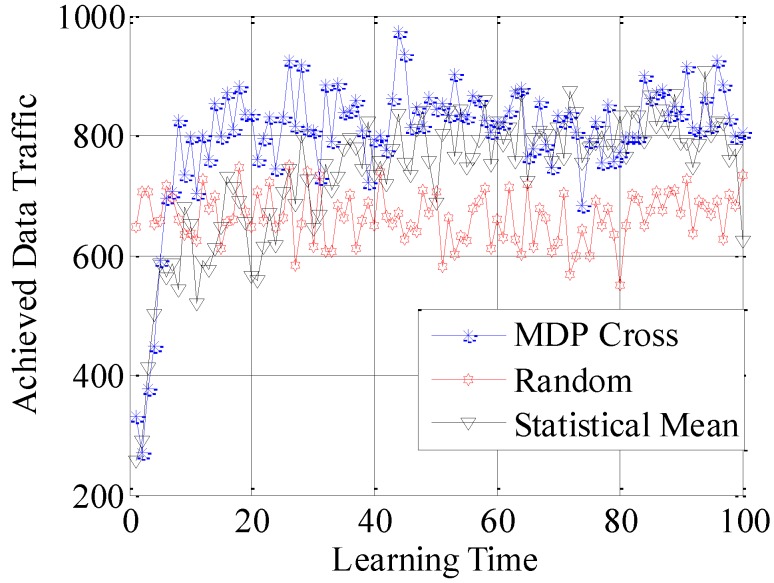
Achieved data traffic of the SUTCH under three strategies with learning time.

**Figure 12:**
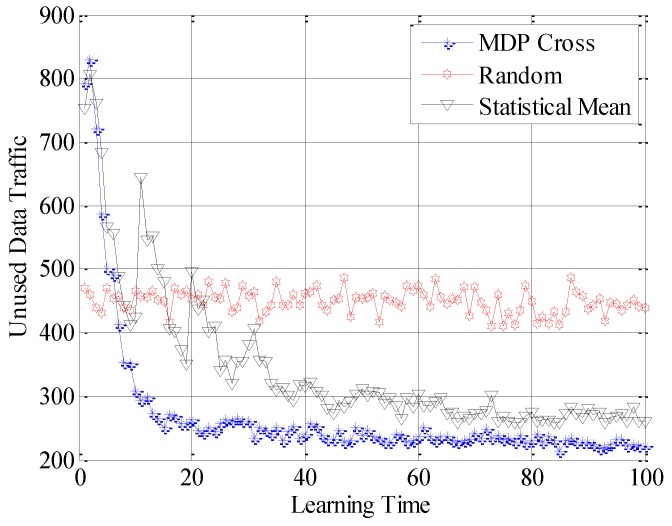
Unused data traffic of the SUTCH under three strategies with learning time.

In Figure 10, Figure 11 and Figure 12, the different performances of the three strategies are shown in detail. Random strategy has the worst accessible performance, because it simply chooses the loading factor Г randomly without proper accessible strategies. Meanwhile, the accessible performance of MDP Cross is better than that of Statistical Mean. Furthermore, the fluctuation of performance curve of MDP Cross is lower than that of Statistical Mean. It is due to the fact that, in the simulation, suppose $\lambda_{m}^{j},$ *j* = 1, 2, …, 6 ∈ [80, 160] the state parameters of the accessible subchannel are changing very fast, therefore, it is a quick-changing subchannel environment. In the quick-changing subchannel environment, the history state information of subchannel environment is changing very fast. However, Statistical Mean will use a lot of history information, so the fast-changing of history information will make a bad influence on choosing the optimal allocation strategy of Г. Therefore, the accessible strategy of MDP Cross fits better in the quick-changing subchannel environment.

**Figure 13:**
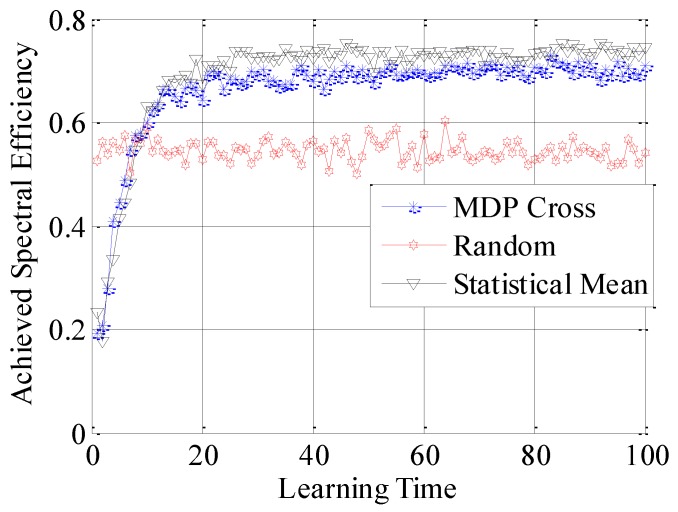
Achieved data traffic of the SUTCH under three strategies with learning time.

**Figure 14:**
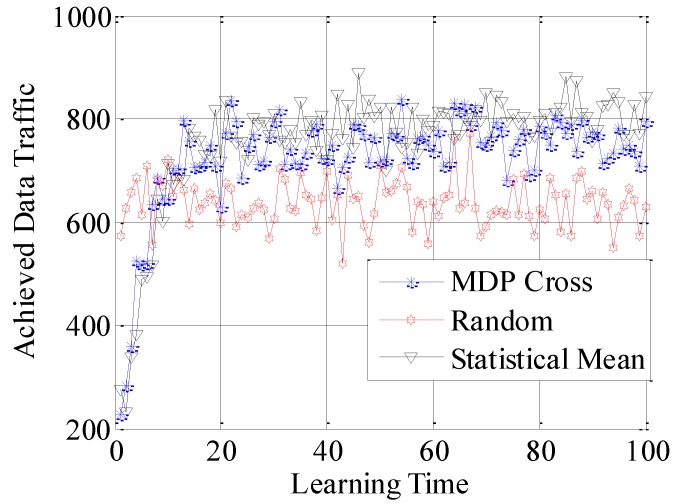
Achieved data traffic of the SUTCH under three strategies with learning time.

**Figure 15:**
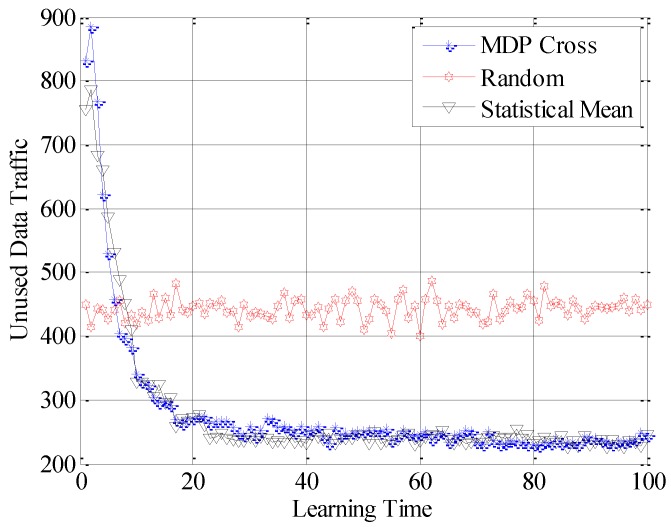
Unused data traffic of the SUTCH under three strategies with learning time.

In the sixth simulation, different performances of the three strategies under constant application scenarios are shown in Figure 13, Figure 14 and Figure 15. Suppose $\lambda_{m}^{j}$ is defined as constant, which is shown as follows:

$$\left[\lambda_{m}^{1},\lambda_{m}^{2},\lambda_{m}^{3},\lambda_{m}^{4},\lambda_{m}^{5},\lambda_{m}^{6}\right]=\left[1/90,1/100,1/110,1/120,1/130,1/140\right]$$

As shown in these figures, the Random strategy still has the worst accessible performance. Meanwhile, the accessible performance of Statistical Mean is better than that of MDP Cross. Furthermore, the fluctuation of the performance curve of Statistical Mean is lower than that of MDP Cross. It is due to the fact that, in a slow-changing subchannel environment, the slow-changing of the history information will have a good influence on choosing the optimal allocation strategy of Г. Therefore, the accessible strategy of Statistical Mean fits better in the slow-changing subchannel environment.

In addition, as shown from Figure 10 to Figure 15, both Statistical Mean and MDP Cross can learn and adapt to the subchannel environment, and converge to a stable state in a short time. Meanwhile, they have the same rate of convergence. According to the analysis in Section 4, both Statistical Mean and MDP Cross have low computation complexity. Therefore, they can be adopted in practice.

## 6. Conclusions

Dynamic spectrum access is an important and necessary technology for future cognitive sensor networks. This paper identified and discussed a new mechanism to set up CR sensor networks without using spectrum holes to convey control information. A transmission channel model was discussed for analyzing the maximum access capacity of different policies and objectives in the fading environment. The maximum achievable capacity of the SUTCH under *ρ*_1_ achieves the poorest performance, since it totally ignores any prior knowledge of the subchannel’s status. When *M*/*N* is small, the best policy for subchannel selection is *ρ_ss_*. In contrast when this ratio is higher, *ρ_ss_* is better.

To solve the trade-off between transmitting power of SUTCH and SUCCH’s capacity, a hybrid access method based on Reinforcement Learning model of MDP Cross and Statistical Mean is also proposed. Both of them outperform the Random strategy, which verified the effectiveness of the proposed methods. In addition, Statistical Mean is more suitable for slow variation application scenarios while MDP Cross performs better in fast variation scenarios.

As is well known, there are many standard structure and policy of reinforcement learning, such as Q-learning and greedy algorithm. Therefore, in the next research, the different learning function and policy should be discussed, which can make a better trade-off between the performance and computation complexity.

## Figures and Tables

**Table 1:** Simulation parameters.

Parameter	Value
*N*	40
Number of active PUs and SUs	[1, 40]
*G*_SUCCH_	2048
Loading factor Г	[1/160, 1/200, ..., 1/400]
Random test times for each Г	750,000

**Table 2:** Simulation parameters.

Parameters	Values
*N*	40
*M*	[0, 6]
Number of the active PUs	34
Number of the SUs	1
*λ_ps_*, *λ_ss_*	1, 1
$\lambda_{m}^{j},$ *j* = 1, 2, …, 6	[80, 160]
*Q*	0.0001 W
*Q*_SUCCH_/*Q*	[0.01, 0.02, ..., 0.1]
*θ_Q_*	−30 dB
*G*	128
*e*	0.01 and 0.05
*∂*_1_, *∂*_2_	0.005, 0.005
*R*	10
*P^n^*(*k*), *n* = [1, *R*]	[1/*R*]
Learning time	100 (times of SUs access)

## References

[1] Zhao Q., Sadler B.M. (2007). A Survey of Dynamic Spectrum Access: Signal Processing, Networking, and Regulatory Policy. IEEE Signal Process. Mag..

[2] Joshi G.P., Nam S.Y., Kim S.W. (2013). Cognitive Radio Wireless Sensor Networks: Applications, Challenges and Research Trends. Sensors.

[3] Liu X. (2015). A Novel Wireless Power Transfer-Based Weighed Clustering Cooperative Spectrum Sensing Method for Cognitive Sensor Networks. Sensors.

[4] Kondareddy Y.R., Agrawal P., Sivalingam K. Cognitive radio network setup without a common control channel. Proceedings of the 2008 IEEE Military Communications Conference (MILCOM 2008).

[5] Baldo N., Asterjadhi A., Zorzi M. (2010). Dynamic spectrum access using a network coded cognitive control channel. IEEE Trans. Wirel. Commun..

[6] Cormio C., Chowdhury K.R. (2015). An Energy-Efficient Spectrum-Aware Reinforcement Learning-Based Clustering Algorithm for Cognitive Radio Sensor Networks. Sensors.

[7] Khoshkholgh M.G., Navaie K., Yanikomeroglu H. (2010). Access strategies for spectrum sharing in fading environment: Overlay, underlay and mixed. IEEE Trans. Mob. Comput..

[8] Gastpar M. (2007). On Capacity under Receive and Spatial Spectrum Sharing Constraints. IEEE Trans. Inf. Theory.

[9] Viterbi A.J. (1995). CDMA: Principles of Spread Spectrum Communication.

[10] Chakravarthy V., Wu Z., Temple M., Garber F. (2009). Novel Overlay/Underlay Cognitive Radio Waveforms Using SD-SMSE Framework to Enhance Spectrum Efficiency—Part I: Theoretical Framework and Analysis in AWGN Channel. IEEE Trans. Commun..

[11] Chakravarthy V., Wu Z., Temple M. (2010). Novel Overlay/Underlay Cognitive Radio Waveforms Using SD-SMSE Framework to Enhance Spectrum Efficiency—Part II: Analysis in Fading Channel. IEEE Trans. Commun..

[12] Jasbi F., So D.K. (2016). Hybrid Overlay/Underlay Cognitive Radio Network with MC-CDMA. IEEE Trans. Veh. Technol..

[13] Su H., Zhang X. (2008). Cross-Layer Based Opportunistic MAC Protocols for QOS Provisioning over Cognitive Radio Wireless Networks. IEEE J. Sel. Areas Commun..

[14] Gupta P., Kumar P.R. (2000). The Capacity of Wireless Networks. IEEE Trans. Inf. Theory.

[15] Tse D., Viswanath P. (2004). Fundamentals of Wireless Communication.

[16] Jafar S.A., Srinivasa S. (2007). Capacity Limits of Cognitive Radio with Distributed and Dynamic Spectral Activity. IEEE J. Sel. Areas Commun..

[17] Ghasemi A., Sousa E.S. (2007). Fundamental Limits of Spectrum Sharing in Fading Environments. IEEE Trans. Wirel. Commun..

[18] Ross S.M. (2012). A First Course in Probability.

[19] Khoshkholgh M.G., Navaie K., Yanikomeroglu H. (2010). Achievable Capacity in Hybrid DS-CDMA/OFDM Spectrum-Sharing. IEEE Trans. Mob. Comput..

[20] Boyd S., Vandenberghe L. (2004). Convex Optimization.

[21] Papoulis A., Pillai S.U. (2002). Probability, Random Variables, and Stochastic Processes.

[22] Kaelbling L.P. (1996). Reinforcement Learning: A Survey. J. Artif. Intell. Res..

[23] Brodersen R.W., Wolisz A., Cabric D., Mishra S.M., Willkomm D. (2004). CORVUS: A Cognitive Radio Approach for Usage of Virtual Unlicensed Spectrum.

[24] Bush R.R., Mosteller F. (1955). Stochastic Models for Learning.

[25] Roth A E., Erev I. (1995). Learning in Extensive Form Games: Experimental Data and Simple Dynamic Models in the Intermediate Run. Games Econ. Behav..

[26] Li J., Bai C., Peng H. (2013). Review of Learning Model and Experiment Based on Learning Theory. Sci. Technol. Manag. Res..

